# Heterogeneous treatment effects of intensive glycemic control on major adverse cardiovascular events in the ACCORD and VADT trials: a machine-learning analysis

**DOI:** 10.1186/s12933-022-01496-7

**Published:** 2022-04-27

**Authors:** Justin A. Edward, Kevin Josey, Gideon Bahn, Liron Caplan, Jane E. B. Reusch, Peter Reaven, Debashis Ghosh, Sridharan Raghavan

**Affiliations:** 1 Division of Cardiology, University of Colorado School of Medicine, Aurora, CO USA; 2 Department of Veterans Affairs Eastern Colorado Healthcare System, Rocky Mountain, Regional VA Medical Center, Medicine Service (111), 1700 North Wheeling Street, Aurora, CO 80045 USA; 3 Department of Biostatistics and Informatics, Colorado School of Public Health, Aurora, CO USA; 4 Department of Veterans Affairs, Hines VA Hospital, Hines, IL USA; 5 Division of Rheumatology, University of Colorado School of Medicine, Aurora, CO USA; 6 Division of Endocrinology, Metabolism, and Diabetes, University of Colorado School of Medicine, Aurora, CO USA; 7 Department of Veterans Affairs Phoenix VA Medical Center, Phoenix, AZ USA; 8 Division of Biomedical Informatics and Personalized Medicine, University of Colorado School of Medicine, Aurora, CO USA; 9 Colorado Cardiovascular Outcomes Research Consortium, Aurora, CO USA

**Keywords:** Machine learning, Glycemic control, Subgroup effects, Heterogeneity, Type 2 diabetes

## Abstract

**Background:**

Evidence to guide type 2 diabetes treatment individualization is limited. We evaluated heterogeneous treatment effects (HTE) of intensive glycemic control in type 2 diabetes patients on major adverse cardiovascular events (MACE) in the Action to Control Cardiovascular Risk in Diabetes Study (ACCORD) and the Veterans Affairs Diabetes Trial (VADT).

**Methods:**

Causal forests machine learning analysis was performed using pooled individual data from two randomized trials (n = 12,042) to identify HTE of intensive versus standard glycemic control on MACE in patients with type 2 diabetes. We used variable prioritization from causal forests to build a summary decision tree and examined the risk difference of MACE between treatment arms in the resulting subgroups.

**Results:**

A summary decision tree used five variables (hemoglobin glycation index, estimated glomerular filtration rate, fasting glucose, age, and body mass index) to define eight subgroups in which risk differences of MACE ranged from − 5.1% (95% CI − 8.7, − 1.5) to 3.1% (95% CI 0.2, 6.0) (negative values represent lower MACE associated with intensive glycemic control). Intensive glycemic control was associated with lower MACE in pooled study data in subgroups with low (− 4.2% [95% CI − 8.1, − 1.0]), intermediate (− 5.1% [95% CI − 8.7, − 1.5]), and high (− 4.3% [95% CI − 7.7, − 1.0]) MACE rates with consistent directions of effect in ACCORD and VADT alone.

**Conclusions:**

This data-driven analysis provides evidence supporting the diabetes treatment guideline recommendation of intensive glucose lowering in diabetes patients with low cardiovascular risk and additionally suggests potential benefits of intensive glycemic control in some individuals at higher cardiovascular risk.

**Supplementary Information:**

The online version contains supplementary material available at 10.1186/s12933-022-01496-7.

## Background

Type 2 diabetes is prevalent and costly, affecting nearly 1 in 10 adults in the United States at an estimated health care cost of $327 billion [1] and affecting over 450 million individuals worldwide [2] Professional society guidelines recognize type 2 diabetes patient heterogeneity through recommendations for individualized treatment of type 2 diabetes patients [3, 4]. Specifically, treatment guidelines suggest consideration of comorbidity burden and age to guide glycemic control intensity and specific medication choice, acknowledging variation in the risks of over- versus under-treatment in different subgroups of type 2 diabetes patients. Real-world data, however, suggests widespread clinical inertia in diabetes care [5–7], potentially reflecting the paucity of evidence to guide treatment individualization. Thus, tools to identify individuals for whom intensive glycemic control may be beneficial are needed, especially for reducing risk of cardiovascular disease, which remains the leading cause of mortality in type 2 diabetes patients [8, 9].

Subgroup analyses of randomized trials may provide the best evidence to guide individualized intensification of glycemic control for cardiovascular risk reduction. The Action to Control Cardiovascular Risk in Diabetes (ACCORD) study and the Veterans Affairs Diabetes Trial (VADT) did not find associations of intensive glycemic control with MACE [10, 11]. However, prior subgroup analyses of both the ACCORD and VADT trials suggest heterogeneous treatment effects (HTE). Individuals without a history of cardiovascular events prior to randomization or whose baseline hemoglobin A1c (HbA1c) was ≤ 8.0% in the ACCORD trial demonstrated a reduction in the primary outcome of MACE in the intensive treatment group [10]. Similarly, intensive glycemic control was associated with reduced cardiovascular events in VADT trial participants with lower coronary artery calcium scores [12].

In contrast to these univariate, hypothesis-driven subgroup analyses, machine learning provides hypothesis-free approaches to evaluating patient subgroups based on combinations of variables [13]. In this study, we aimed to address evidence gaps for type 2 diabetes treatment individualization to mitigate cardiovascular disease risk using a hypothesis-free, data-driven method—causal forests machine learning [14–16]—to identify HTE of intensive glycemic control on MACE in the ACCORD and VADT studies.

## Materials and methods

### Study samples

The Colorado Multiple Institutional Review Board and local VA Research and Development Committee provided human subjects oversight and approval of the study. We included individual-level data from two randomized clinical trials in this study. The ACCORD and VADT studies have been described in detail previously [10, 11]. Both studies included adults with type 2 diabetes and a hemoglobin A1c (HbA1c) ≥ 7.5% at enrollment. The VADT study enrolled participants from December 2000 through May 2003, and follow-up continued through May 2008; median follow-up time in the VADT study was 5.6 years. The ACCORD study enrolled participants at high cardiovascular risk from January 2001 to October 2005; follow-up in the ACCORD study continued until June 2009 with a median on-protocol follow-up time of 3.7 years and a median total follow-up time of 4.9 years. Both studies randomized participants to receive intensive or standard glycemic control. The VADT study aimed to achieve a target of at least 1.5% lower HbA1c in participants randomized to intensive control compared to standard control. In the ACCORD study, intensive glycemic control participants were treated to a target HbA1c < 6% as compared to a target HbA1c of 7–7.9% for the standard glycemic control arm. In this secondary analysis, we included data from all 1791 VADT study participants and 10,251 ACCORD study participants.

### Outcome

The primary outcome was major adverse cardiovascular events, defined as fatal or non-fatal myocardial infarction or stroke based on endpoint adjudication in the original trials. All-cause mortality was a secondary outcome.

### Predictors

We included baseline variables that were common to the two studies: patient demographics, comorbidities, diabetes medications, cardiovascular disease medications, and laboratory values (Table 1; Additional file 1 Table S1). Estimated glomerular filtration rate (eGFR) was calculated using the Modification of Diet in Renal Disease Study Equation [17]. Hemoglobin glycation index (HGI) was estimated as the residual between measured HbA1c and HbA1c predicted by regressing on fasting glucose in the ACCORD study participants [18].

**Table 1:** Study population characteristics

	ACCORD		VADT		ACCORD + VADT	
	Standard control	Intensive control	Standard control	Intensive control	Standard control	Intensive control
	N = 5123	N = 5128	N = 899	N = 892	N = 6022	N = 6020
Age, mean (SD)	62.8 (6.7)	62.8 (6.6)	60.3 (8.6)	60.5 (8.8)	62.4 (7.0)	62.4 (7.0)
Sex, n female (%)	1969 (38.4)	1983 (38.7)	26 (2.9)	26 (2.9)	1995 (33.1)	2009 (33.4)
Race, n (%)						
Black	956 (18.7)	997 (19.4)	147 (16.4)	152 (17.0)	1103 (18.3)	1149 (19.1)
Hispanic	379 (7.4)	358 (7.0)	136 (15.1)	155 (17.4)	515 (8.6)	513 (8.5)
HbA1c (%), mean (SD)	8.3 (1.1)	8.3 (1.1)	9.4 (1.6)	9.4 (1.5)	8.5 (1.2)	8.5 (1.2)
Glucose (mg/dL), mean (SD)	175.7 (56.4)	174.7 (55.9)	205.9 (69.0)	203.5 (67.8)	180.2 (59.5)	179.0 (58.7)
Hgb glycation index (unitless), mean (SD)	− 0.07 (0.9)	− 0.08 (1.0)	0.8 (1.4)	0.8 (1.4)	0.06 (1.1)	0.05 (1.1)
Total cholesterol (mg/dL), mean (SD)	183.3 (41.6)	183.3 (42.1)	184.7 (52.7)	181.6 (40.4)	183.5 (43.5)	183.1 (41.8)
Triglycerides (mg/dL), mean (SD)	189.4 (148.6)	190.9 (148.2)	222.8 (351.8)	200.8 (161.8)	194.4 (193.5)	192.4 (150.3)
LDL cholesterol (mg/dL), mean (SD)	104.9 (33.8)	104.9 (34.0)	108.2 (34.0)	107.0 (30.9)	105.4 (33.9)	105.2 (33.6)
HDL cholesterol (mg/dL), mean (SD)	41.9 (11.5)	41.8 (11.8)	35.8 (10.4)	36.2 (9.9)	41.0 (11.5)	41.0 (11.7)
Creatinine (mg/dL), mean (SD)	0.9 (0.2)	0.9 (0.2)	1.0 (0.2)	1.0 (0.2)	0.9 (0.2)	0.9 (0.2)
eGFR (mL/min/1.73m^2^), mean (SD)	91.3 (28.4)	90.8 (25.8)	87.5 (22.6)	87.3 (24.2)	90.7 (27.7)	90.3 (25.6)
ALT (mg/dL), mean (SD)	27.7 (14.9)	27.5 (17.4)	31.9 (17.4)	30.8 (15.2)	28.3 (15.3)	28.0 (17.1)
SBP (mmHg), mean (SD)	136.5 (17.2)	136.2 (17.0)	131.8 (16.8)	131.4 (16.6)	135.8 (17.2)	135.5 (17.1)
DBP (mmHg), mean (SD)	75.0 (10.7)	74.8 (10.7)	76.1 (10.2)	76.0 (10.4)	75.2 (10.6)	75.0 (10.6)
BMI (kg/m^2^), mean (SD)	32.2 (5.4)	32.2 (5.4)	31.2 (4.4)	31.3 (4.4)	32.1 (5.3)	32.1 (5.3)
Diabetes duration (years), mean (SD)	10.9 (7.6)	10.7 (7.6)	11.5 (7.2)	11.5 (7.8)	11.0 (7.6)	10.9 (7.6)
Insulin use, n (%)	1832 (35.8)	1750 (34.1)	467 (51.9)	466 (52.2)	2299 (38.2)	2216 (36.8)
Sulfonylurea use, n (%)	2707 (52.9)	2767 (54.0)	561 (62.4)	529 (59.3)	3268 (54.3)	3296 (54.8)
Metformin use, n (%)	3285 (64.1)	3269 (63.7)	632 (70.3)	605 (67.8)	3917 (65.1)	3874 (64.4)
Glinide use, n (%)	131 (2.6)	126 (2.5)	4 (0.4)	5 (0.6)	135 (2.2)	131 (2.2)
Acarbose use, n (%)	45 (0.9)	50 (1.0)	16 (1.8)	20 (2.2)	61 (1.0)	70 (1.2)
Thiazolidinedione use, n (%)	1125 (22.0)	1133 (22.1)	171 (19.0)	166 (18.6)	1296 (21.5)	1299 (21.6)
History of amputation, n (%)	106 (2.1)	111 (2.2)	27 (3.0)	28 (3.1)	133 (2.2)	139 (2.3)
History of eye surgery, n (%)	1169 (22.9)	1119 (21.9)	150 (18.3)	152 (18.9)	1319 (22.3)	1271 (21.5)
Current smoker, n (%)	607 (11.8)	640 (12.5)	145 (16.2)	154 (17.3)	752 (12.5)	794 (13.2)
History of MI, n (%)	803 (15.7)	787 (15.3)	170 (19.0)	166 (18.6)	973 (16.2)	953 (15.8)
History of stroke, n (%)	325 (6.3)	305 (5.9)	41 (4.6)	56 (6.3)	366 (6.1)	361 (6.0)
History of CHF, n (%)	245 (4.8)	249 (4.9)	48 (5.3)	61 (6.8)	293 (4.9)	310 (5.2)
History of angina, n (%)	560 (10.9)	608 (11.9)	166 (18.5)	167 (18.7)	726 (12.1)	775 (12.9)
Prior coronary revascularization, n (%)	556 (10.9)	615 (12.0)	183 (20.4)	182 (20.4)	739 (12.3)	797 (13.2)

*ACCORD* action to control cardiovascular risk in diabetes study, *VADT* veterans affairs diabetes trial, *HbA1c* hemoglobin A1c, *DBP* diastolic blood pressure, *SBP* systolic blood pressure, *eGFR* estimated glomerular filtration rate, *BMI* body mass index, *ALT* alanine amino transferase, *HDL* high-density lipoprotein, *LDL* low-density lipoprotein, *MI* myocardial infarction, *CHF* congestive heart failure

### Statistical approach

We fit causal forests [14–16] to identify HTE of intensive glycemic control in the ACCORD and VADT studies. We first examined ACCORD and VADT studies separately and report the correlation between variable importance statistics from the two causal forests using Kendall’s tau b. We then fit another causal forest using pooled individual-level data from both studies. Finally, we used variable importance from the causal forest analysis of the pooled study data to construct a representative causal tree. All analyses with causal forests contained 5000 trees and a minimum node size of approximately 5% of the total sample size, with each tree fit using an honest splitting and estimation approach [14–16] from random samples representing half of the stratified samples. To avoid overfitting, each tree only considers half of the covariates for splitting, randomly selected from the set of predictors. To compare contributions of variables to HTE, we employed a statistic included in the *grf* package in R which generates a weighted average of importance for each variable [15, 16].

As there is not a consensus approach for translating causal forests to decision trees, we employed an approach that we have previously used to generate a summary decision tree from causal forests [19]. Specifically, we used the variable importance measure to identify the top variables contributing to HTE based on causal forests, which we then use to build a summary decision tree with the caveat that this approach will not always identify significant HTE subgroups in the data [15]. We found that a stable summary causal tree with eight subgroups based on five of the top eight variables resulted when we included 8, 9, or 10 of the most important variables based on causal forests analysis of pooled study data. The summary causal tree once again required at least 5% of the total sample in every terminal node, and honest cross-validation for tuning the shrinkage parameter [15]. To quantify HTE in the summary causal tree, we calculated the absolute risk difference in MACE (primary outcome) and all-cause mortality (secondary outcome) between the intensive and standard glycemic control arms within each terminal node subgroup of the summary causal tree using pooled data from both trials and in the ACCORD and VADT samples separately. We plotted cumulative incidence of MACE in the intensive and standard glycemic control arms using pooled data from both the ACCORD and VADT studies in each of the subgroups of the summary causal tree and compared the incidence curves using a log-rank test.

All analyses were conducted in R (version 3.5.3, R Foundation for Statistical Computing, Vienna, Austria). Statistical code is available upon request.

## Results

In comparison to the VADT study population, the ACCORD study group had a larger proportion of women, but a smaller proportion of participants with Hispanic ethnicity. ACCORD study participants had a lower HbA1c and were less likely to use insulin. The VADT study population included more participants with a history of angina, prior history of MI and congestive heart failure, and prior coronary artery revascularization. Additional similarities and differences in baseline patient characteristics can be found in Table 1.

While several variables were highly ranked both when causal forests were applied to the ACCORD study and to the VADT study separately, variable importance ranks were only moderately correlated (Kendall’s tau-b of 0.632; Additional file 1: Fig. S1). Next, we repeated the causal forests analysis using pooled data from both studies and including an indicator variable for study (ACCORD or VADT). Out of 47 variables evaluated, the ten most highly prioritized variables (HGI, fasting glucose, diabetes duration, total cholesterol, high-density lipoprotein cholesterol, eGFR, BMI, age, low-density lipoprotein cholesterol, and HbA1c) after applying causal forests to the pooled study data are shown in Additional file 1: Table S2. Of these top variables, most were also among the most highly prioritized variables when performing the same analysis on each individual study (Additional file 1: Table S2). Notably, the study indicator variable had an importance score of 0.00.

**Fig. 1:**
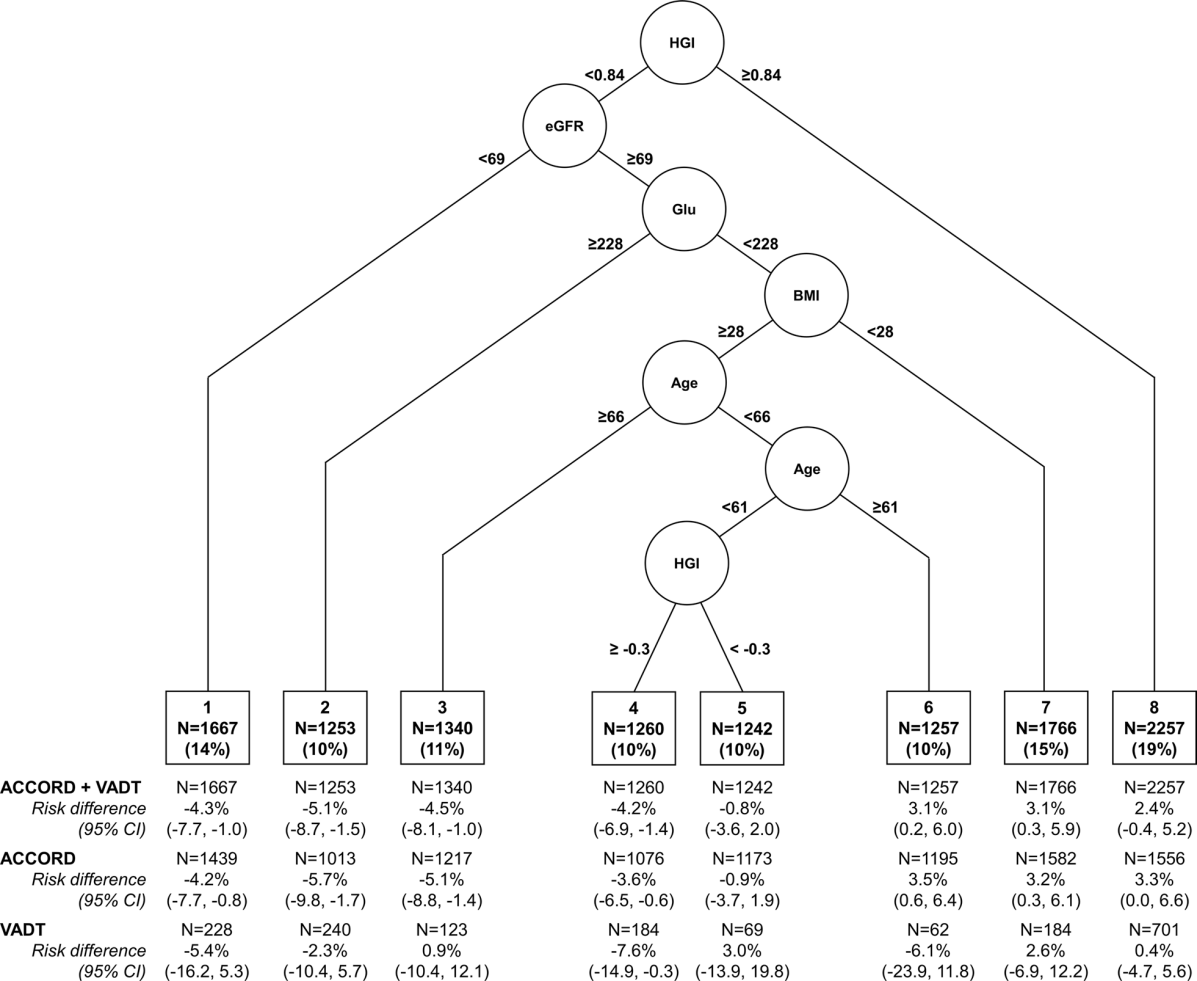
Summary causal tree of heterogeneous treatment effects of intensive glycemic control on all-cause mortality. Splitting variables and cut-points for each split are shown, resulting in eight terminal subgroups (N (%) represent number and proportion of participants in pooled ACCORD + VADT sample in each subgroup). Units for splitting variables are mL/min for eGFR (estimated glomerular filtration rate), mg/dL for serum glucose, kg/m^2^ for BMI (body-mass index), and years for age. Risk difference of MACE and 95% confidence intervals in each subgroup in the pooled sample and in each study alone shown below diagram

**Fig. 2:**
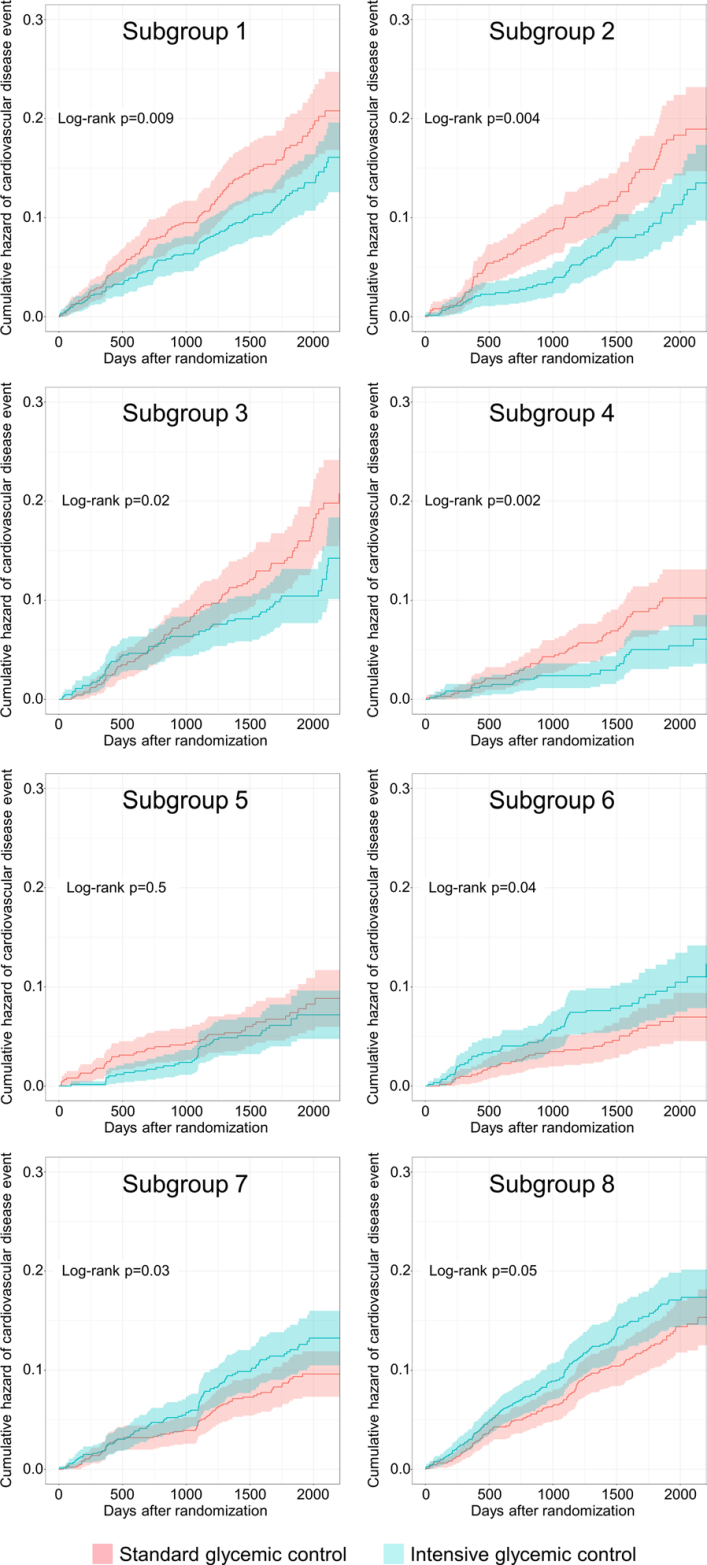
Cumulative incidence curves for major adverse cardiovascular events within each subgroup identified by causal forests applied to pooled data from both the ACCORD and VADT studies. Cumulative incidence is represented by the lines with 95% confidence intervals indicated by the shaded regions with standard glycemic control arm in pink and intensive glycemic control arm in green for each subgroup

We next generated a summary causal tree that defined specific HTE subgroups. The summary causal tree was stable when including 8–10 of the most highly prioritized variables from the causal forest analysis of the pooled ACCORD and VADT study data, utilizing only five variables to divide the pooled sample into eight subgroups (Fig. 1). In subgroups 1–4, comprising 45% of the pooled sample, intensive glycemic control was associated with lower MACE (risk difference of − 4.3% [95% CI: − 7.7, − 1.0], − 5.1% [95% CI: − 8.7, − 1.5], − 4.5% [95% CI: − 8.1, − 1.0], and − 4.2% [95% CI: − 6.9, − 1.4], respectively; Fig. 1), and lower cumulative incidence of MACE over the follow-up time (Fig. 2). Subgroup 4 comprising 10% of the pooled sample also demonstrated consistent direction of effect and 95% confidence intervals excluding the null with intensive glycemic control associated with lower incidence of MACE in both the ACCORD and VADT studies (risk difference − 3.6% [95% CI: − 6.5, − 0.6] in ACCORD and − 7.6% [95% CI: − 14.9, − 0.3] in VADT). In two subgroups (subgroups 6 and 7) intensive glycemic control was associated with higher MACE in the pooled sample (risk difference of 3.1% [95% CI: 0.2, 6.0] and 3.1% [95% CI: 0.3, 5.9], respectively; Fig. 1), and with higher cumulative incidence of MACE over the follow-up time (Fig. 2). Neither subgroup 6 nor 7 exhibited consistent and significant associations of intensive glycemic control with higher MACE in the ACCORD and VADT study samples separately. The direction of effect of intensive glycemic control on MACE in Subgroup 7 was consistent in both study samples but with 95% confidence intervals including the null in VADT (3.2% [95% CI: 0.3, 6.1] in ACCORD, and 2.6% [95% CI: − 6.9, 12.2] in VADT; Fig. 1).

**Fig. 3:**
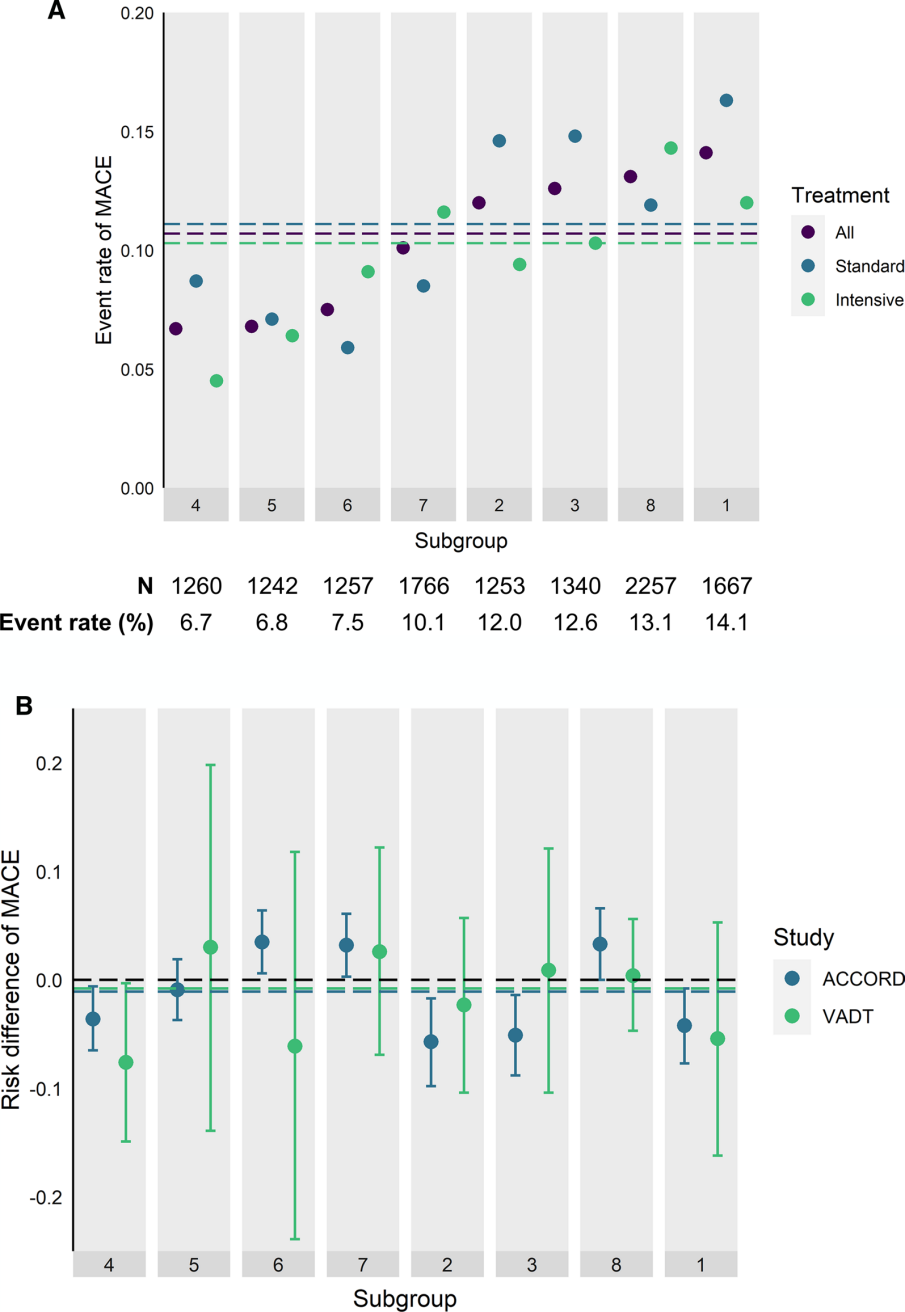
Comparison of subgroup effects based on subgroup-specific rates of major adverse cardiovascular events (MACE). Subgroups were ordered from left to right by event rates in pooled data including both ACCORD and VADT studies. **A** Event rates in each subgroup across both treatment arms (“All”, purple), among those randomized to standard glycemic control (“Standard”, blue), and among those randomized to intensive glycemic control (“Intensive”, green). Dotted lines show the event rates of MACE in the full sample (purple), in those randomized to standard glycemic control (blue), and in those randomized to intensive glycemic control (green). **B** Risk differences of MACE associated with standard versus intensive glycemic control, stratified by study and subgroup with subgroups ordered from left to right by increasing MACE event rates in the full sample including pooled data from both studies. Positive risk differences reflect higher MACE and negative risk differences reflect lower MACE in intensive glycemic control compared to standard glycemic control. Blue and green dotted lines represent the average treatment effect of intensive versus standard glycemic control on MACE in the ACCORD and VADT studies, respectively

As HTE can be a function of absolute event rates [20], we examined whether subgroup-specific effects of intensive glycemic control on MACE correlated with subgroup-specific MACE rates. Subgroup 4, in which intensive glycemic control was associated with lower MACE in pooled analysis of the ACCORD and VADT studies and when each study was examined separately, had the lowest MACE rate of the eight subgroups identified by causal forests (Fig. 3). We did not observe a discernible pattern in HTE in relation to increasing MACE rates across subgroups. In fact, intensive glycemic control was associated with lower MACE in both the subgroup with the lowest event rate (Subgroup 4) and the subgroup with the highest event rate (Subgroup 1) (Fig. 3; Additional file 1: Fig S2).

To determine if any beneficial effects of intensive glycemic control on MACE were balanced by detrimental effects on mortality, we examined all-cause mortality associated with intensive glycemic control in the 8 subgroups identified in the summary causal tree for HTE on MACE. In subgroup 4—in which intensive glycemic control was associated with lower MACE in pooled data analysis and in each trial separately—intensive glycemic control was not associated with all-cause mortality (risk difference of − 0.8% [95% CI: − 2.8, 1.2] in pooled sample, − 1.0% [95% CI: − 3.2, 1.2] in ACCORD, and 0.5% [95% CI: − 4.7, 5.7] in VADT; (Additional file 1: Table S3). Intensive glycemic control, however, was associated with higher all-cause mortality in subgroup 8 in analysis of pooled data from both trials and in ACCORD study data alone (Additional file 1: Table S3), confirming the identification of HGI in prior work as a determinant of HTE of intensive glycemic control on all-cause mortality [13]. None of the other subgroups exhibited significant associations of glycemic control intensity with all-cause mortality (Additional file 1: Table S3).

## Discussion

In this secondary analysis of the ACCORD and VADT trials, we found heterogeneous treatment effects of intensive glycemic control on MACE. The most influential variables for identifying HTE are factors known to be associated with either cardiovascular disease or diabetes-related outcomes. A summary causal tree using the top variables from causal forests applied to pooled data from both trials defined eight HTE subgroups. Three subgroups (34% of the combined ACCORD and VADT sample) had consistent associations of intensive glycemic control with cardiovascular benefit in pooled data and in ACCORD and VADT separately, and two subgroups (34% of the combined ACCORD and VADT sample) demonstrated worse cardiovascular outcomes associated with intensive glycemic control in pooled data and in ACCORD and VADT separately.

We did not observe a consistent pattern of cardiovascular benefit or harm of intensive glycemic control in relation to cardiovascular risk in the subgroups. One subgroup (Subgroup 4) demonstrated lower MACE associated with intensive glycemic control in the pooled sample, the ACCORD trial, and the VADT trial samples. Aside from a BMI ≥ 28 kg/m^2^, this was a relatively healthy subgroup of trial participants: age < 61 years, glucose ≤ 228 mg/dL, eGFR ≥ 69 mL/min/1.73m^2^, and low HGI bounded between − 0.3 and 0.84. Consistent with these clinical characteristics, this subgroup had the lowest rate of MACE in the pooled sample of the ACCORD and VADT studies. Moreover, intensive glycemic control was not associated with all-cause mortality in this subgroup across both trials, providing reassurance that cardiovascular disease risk reduction was not offset by higher non-cardiovascular mortality. The identification of this subgroup lends supportive evidence to current treatment guidelines, which suggest that intensive glycemic control targets may be considered in diabetes patients who are younger with few medical comorbidities [3, 4].

On the other hand, our analysis also identified lower MACE associated with intensive glycemic control in Subgroup 1, the subgroup with the highest risk of cardiovascular events. We observed directional consistency of the HTE in Subgroup 1 in the ACCORD and VADT study samples separately, though with wide confidence intervals that include the null in VADT. Similarly, Subgroups 6, 7, and 8, which all exhibited higher MACE associated with intensive glycemic control in the pooled analysis of VADT and ACCORD, were at low-, intermediate-, and high-risk of MACE, respectively, in the pooled study sample. While HTE are often correlated with underlying risk of the outcome—forming the basis for risk-based treatment recommendations in many clinical contexts [21, 22], the approach used here identified HTE of intensive glycemic control that appear independent of MACE risk. Thus, machine learning approaches may provide complementary information to cardiovascular risk estimation to guide diabetes treatment individualization across the spectrum of cardiovascular disease risk.

Our finding that HGI was the most highly ranked variable in the analysis of pooled data from the ACCORD and VADT trials extends prior work describing associations of glycemic variability with cardiovascular risk in diabetes patients. As mentioned above, HGI is the difference between measured HbA1c and the HbA1c that would be predicted on the basis of a concomitant fasting plasma glucose measurement. A high HGI, therefore, would indicate that an individual’s HbA1c is higher than would be predicted from fasting plasma glucose, potentially implying high glucose variability not reflected in the fasting glucose measurement. The identification of high HGI as an important determinant of adverse cardiovascular effects of intensive glycemic control (Subgroup 8) supports prior work that has found associations of glucose and HbA1c variability with microvascular and macrovascular complications and hypoglycemia in diabetes patients, including in the VADT and ACCORD trials [10, 23–32]. While calculating HGI in routine care may be impractical as it is derived by regressing HbA1c on glucose in a population, our findings and the prior work on glycemic variability suggest that discordance between glycemia measured by HbA1c and fasting glucose may be a useful adverse prognostic indicator. When assessing risk of microvascular complications, initial work from McCarter et al. concluded that HGI was an independent predictor of risk [33]. A follow up study by Lachlin et al. however, argued that HGI was highly correlated with the HbA1c level, and that it is not an independent predictor of the risk of microvascular complications [34]. Their conclusion was that the effect of the glycation index on risk can be explained by the associated level of HbA1c. While others have explored comparative risk of outcomes related to HbA1C and HGI, the prioritization of HGI more highly than HbA1c when including both variables in our analysis may suggest that HGI captures treatment-related risk in ways that are not redundant with HbA1c.

Although we found one subgroup, Subgroup 4, in which intensive glycemic control was associated with fewer cardiovascular events, we would not interpret this result as advocating for treating similar real-world patients to an HbA1c target < 6%. That a similar benefit was observed in the VADT study and in the ACCORD study—which had very different HbA1c targets—suggests that a more intensive glycemic control strategy may be beneficial for certain patients without targeting specific HbA1c thresholds. Given mounting evidence of the efficacy of glucagon-like peptide-1 receptor agonists and sodium glucose co-transporter-2 inhibitors for improving cardiovascular outcomes in patients with diabetes independent of effects on glycemia [35–40], optimal cardiovascular disease prevention through diabetes treatment may depend on both individualized glycemic control goals and medication choice. Future work examining HTE of the new cardioprotective classes of diabetes medications may identify evidence-based strategies for tailoring diabetes treatment based on more than just underlying cardiovascular risk.

Identifying subpopulations of type 2 diabetes patients has been of great interest over the last several years with analyses focused on understanding predictors of disease progression and treatment response. Our analysis complements prior hypothesis-free, data-driven analyses [41, 42] and hypothesis-based analysis using clinical features [43] to define diabetes patient subgroups with differential response to treatment. Prior secondary analyses of the ACCORD study suggest that features of on-trial HbA1c may be associated with trial outcomes [42, 44]– an approach that contrasts with our study focused on baseline characteristics in which HbA1c was not used in the summary decision tree. In addition to highlighting the urgent need to understand how diabetes patient heterogeneity might inform better tailoring of treatment, our study draws attention to important data and methodological gaps in advancing diabetes precision medicine. First, causal forests applied to each of the trials separately yielded only modest correlation in variable importance rankings, highlighting the value of pooling individual-level data from multiple studies when examining HTE using machine-learning and the potential sensitivity of these methods to between-study heterogeneity in study populations and intervention design [19]. Differences in the trial designs of ACCORD (factorial) [10] and VADT (parallel treatment RCT) [11] may also have contributed to differences in results when analyzing the trials separately. To make the most of trial data, methods that can flexibly accommodate differences in trial design are needed. Second, there is a lack of consensus on how best to translate causal forests results to interpretable, mutually exclusive subgroups of real-world patients. In this study we used the most highly prioritized variables from causal forests to generate a summary decision tree, an approach that can improve interpretability of the causal forests output but can also lead to overfitting. Given the substantial heterogeneity of type 2 diabetes patients, methodological advances in translating machine learning methods to clinical decision-making may prove impactful to individualized diabetes care.

There are several limitations to acknowledge in this study. First, the interpretation of results should remain limited to the populations represented in the ACCORD and VADT studies. Though pooling data from the two studies broadens the general population representation in our analysis, the resulting pooled sample is derived from select randomized trial samples and does not necessarily represent the real-world diabetes patient population. Additional evaluation is needed to assess the performance of the HTE subgroups in the summary causal tree in diabetes patients drawn from the general population and the VA health system. Second, the summary causal tree presented here is difficult to interpret from the perspective of relating variable cut-points to physiology and clinical outcomes and would be difficult to institute into clinical practice in its current form. Third, the VADT study was considerably smaller than the ACCORD study, limiting statistical power for subgroup analyses in the VADT study—reflected by wide confidence intervals for the treatment effects—and granting greater weight to the ACCORD study in pooled analyses. Finally, we evaluated only one machine-learning algorithm for HTE detection—causal forests—in our analysis. Assessing whether similar variables are prioritized for HTE identification across disparate algorithms exceeds the scope of this manuscript but may be a valuable next step in translating results of machine learning subgroup analyses to clinical care.

## Conclusions

In sum, using data from two randomized trials of intensive glycemic control in type 2 diabetes patients, we found subgroups defined by combinations of HGI, eGFR, serum glucose, BMI, and age that exhibited different associations of intensive glycemic control with major adverse cardiovascular events. This hypothesis-free, data driven approach identified a subset of patients consisting of younger trial participants with overweight/obesity, low HGI, preserved renal function, and lower serum glucose levels that may benefit from intensive glycemic control to lower MACE, consistent with contemporary guidelines for the care of diabetes patients. We also highlight that potential benefit of intensive glycemic control to lower MACE was not clearly correlated with underlying risk of MACE in subgroups, suggesting that clinical decision-making for diabetes treatment intensity based primarily on cardiovascular risk estimation may miss patient subgroups at high cardiovascular risk who might benefit from intensive glycemic control.

## Supplementary Information

**Additional file 1: Table S1. **Baseline predictor variables common to VADT and ACCORD studies included in causal forest analysis. **Table S2**. Variable importance ranks for cardiovascular disease events and heterogeneous treatment effects across ACCORD, VADT, and pooled samples. **Figure S1**. Correlation of variable importance rank from causal forest in the VADT and ACCORD studies. **Figure S2. **Comparison of subgroup effects based on subgroup-specific rates of major adverse cardiovascular events (MACE) stratified by study. **Table S3.** Risk differences of mortality between intensive and standard glycemic control arms in in eight subgroups.

## Data Availability

VADT study data were made available through a Data Use Agreement with the VA Cooperative Studies Program (clinical trials registration number NCT00032787). ACCORD study data is publicly available through the US National Institutes of Health, National Heart, Lung, and Blood Institute’s Biologic Specimen and Data Repository Information Coordinating Center (https://biolincc.nhlbi.nih.gov/studies/accord/).

## Abbreviations

ACCORD: Action to Control Cardiovascular risk in Diabetes Study (ACCORD)
BMI: Body-mass index
eGFR: Estimated glomerular filtration rate
HbA1C: Hemoglobin A1c
HGI: Hemoglobin glycation index
HTE: Heterogeneous treatment effects
MACE: Major adverse cardiovascular events
VADT: Veterans Affairs Diabetes Trial

## Declarations

### Study group members

All of the contributing investigators to the Veterans Affairs Diabetes Trial are acknowledged in a document for publication as an online-only supplement.
