# Supplementary material for: Heterogeneous treatment effects of intensive glycemic control on major adverse cardiovascular events in the ACCORD and VADT trials: a machine-learning analysis
Source: Cardiovasc Diabetol. 2022 Apr 27;21:58. doi: 10.1186/s12933-022-01496-7 (PMC9047276; doi:10.1186/s12933-022-01496-7)
Supplement: Supplementary file 1 — Additional file 1: Table S1. Baseline predictor variables common to VADT and ACCORD studies included in causal forest analysis. Table S2. Variable importance ranks for cardiovascular disease events and heterogeneous treatment effects across ACCORD, VADT, and pooled samples. Figure S1. Correlation of variable importance rank from causal forest in the VADT and ACCORD studies. Figure S2. Comparison of subgroup effects based on subgroup-specific rates of major adverse cardiovascular events (MACE) stratified by study. Table S3. Risk differences of mortality between intensive and standard glycemic control arms in in eight subgroups. [file 12933_2022_1496_MOESM1_ESM.docx]

**Heterogeneous treatment effects of intensive glycemic control on major adverse cardiovascular events in the ACCORD and VADT trials: a machine-learning analysis**

Justin A. Edward, Kevin Josey, Gideon Bahn, Liron Caplan, Jane E.B. Reusch, Peter Reaven, Debashis Ghosh, Sridharan Raghavan

**Online Supplementary Material**

**Supplemental Table 1.** Baseline predictor variables common to VADT and ACCORD studies included in causal forest analysis.

**Supplemental Table 2.** Variable importance ranks for cardiovascular disease events and heterogeneous treatment effects across ACCORD, VADT, and pooled samples.

**Supplemental Figure 1.** Correlation of variable importance rank from causal forest in the VADT and ACCORD studies.

**Supplemental Figure 2.** Comparison of subgroup effects based on subgroup-specific rates of major adverse cardiovascular events (MACE) stratified by study.

**Supplemental Table 3.** Risk differences of mortality between intensive and standard glycemic control arms in in eight subgroups.

**List of Contributors to VADT Study.**

**Supplemental Table 1.** Baseline predictor variables common to VADT and ACCORD studies included in causal forest analysis.

| Demographics  Age  Sex  Race – Black  Race – Hispanic |
| --- |
| Blood pressure  Systolic blood pressure  Diastolic blood pressure |
| Laboratory values  ALT  Creatinine  Estimated GFR  Glucose  HbA1c  HDL  LDL  Triglycerides  Total cholesterol |
| Cardiovascular risk factors and history  History of amputation  History of angina  History of myocardial infarction  History of stroke  History of congestive heart failure  History of coronary revascularization  History of percutaneous revascularization  BMI  Smoker |
| Diabetes history  Diabetes duration  History of eye surgery |
| Diabetes medications  Metformin use  Sulfonylurea use  Thiazolidinedione use  Insulin use  Acarbose use  Glinide use |
| Blood pressure medications  ACE inhibitor use  ARB use  Antiadrenergic medication use  Beta blocker use  Calcium channel blocker use  Loop diuretic use  Potassium-sparing diuretic use  Thiazide diuretic use  Other hypertension medication use |
| Other medications  Antiplatelet agent use  Statin use  Fibrates use |
| Other variables  Hemoglobin glycation index (HGI)  Treatment arm  Study (VADT or ACCORD) |

**Supplemental Table 2.** Variable importance ranks for cardiovascular disease events and heterogeneous treatment effects across ACCORD, VADT, and pooled samples.

| **Variable** | **ACCORD + VADT** | | **ACCORD** | | **VADT** | |
| --- | --- | --- | --- | --- | --- | --- |
|  | **Score** | **Rank** | **Score** | **Rank** | **Score** | **Rank** |
| **HGI** | 0.14 | 1 | 0.195 | 1 | 0.038 | 9 |
| **Fasting glucose** | 0.102 | 2 | 0.064 | 6 | 0.079 | 4 |
| **Duration of diabetes (yrs)** | 0.09 | 3 | 0.031 | 14 | 0.163 | 1 |
| **Total cholesterol** | 0.072 | 4 | 0.043 | 11 | 0.082 | 3 |
| **eGFR** | 0.068 | 5 | 0.065 | 5 | 0.056 | 8 |
| **BMI** | 0.059 | 6 | 0.070 | 4 | 0.019 | 16 |
| **HDL cholesterol** | 0.058 | 7 | 0.082 | 2 | 0.028 | 15 |
| **Age** | 0.054 | 8 | 0.080 | 3 | 0.079 | 5 |
| **LDL cholesterol** | 0.050 | 9 | 0.048 | 10 | 0.063 | 7 |
| **HbA1c** | 0.050 | 10 | 0.054 | 8 | 0.029 | 13 |

**Abbreviations**: ACCORD, Action to Control Cardiovascular Risk in Diabetes Study; VADT, Veterans Affairs Diabetes Trial; HGI, hemoglobin glycation index; eGFR, estimated glomerular filtration rate; BMI, body mass index; HDL, high-density lipoprotein; LDL, low-density lipoprotein; HbA1c, hemoglobin A1c

**Supplemental Figure 1.** Correlation of variable importance rank from causal forest in the VADT and ACCORD studies.

**
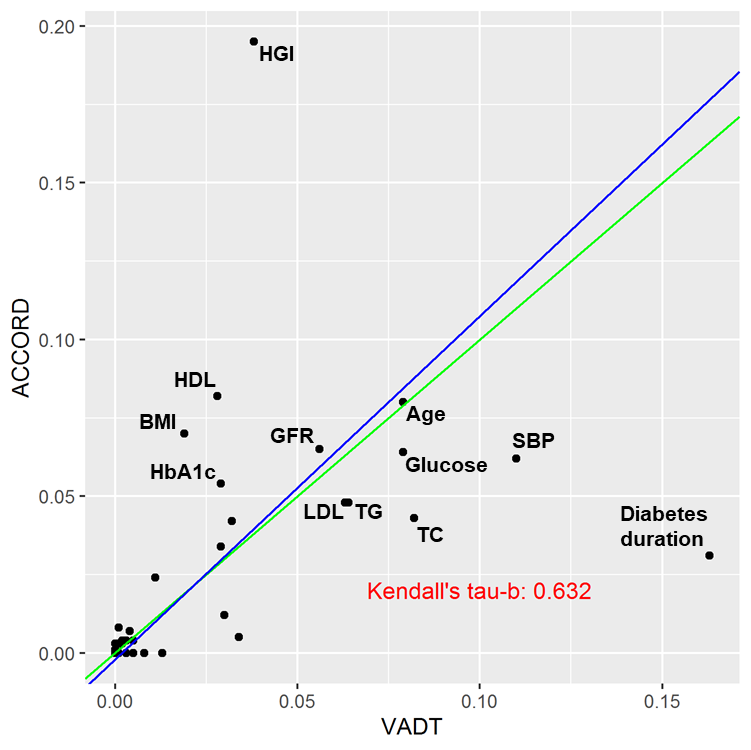
**

**Supplemental Figure 1.** Correlation of variable importance rank from causal forest in the VADT and ACCORD studies. Variable importance score in VADT study on x-axis and in ACCORD study on y-axis. Perfect correlation represented by green line; actual correlation represented by blue line. Key variables indicated (HGI, hemoglobin glycation index; HDL, high-density lipoprotein cholesterol; BMI, body mass index; HbA1c, hemoglobin A1c; eGFR, estimated glomerular filtration rate; LDL, low-density lipoprotein cholesterol; TG, triglycerides; age; glucose; TC, total cholesterol; SBP, systolic blood pressure; diabetes duration).

**Supplemental Figure 2.** Comparison of subgroup effects based on subgroup-specific rates of major adverse cardiovascular events (MACE) stratified by study.

**
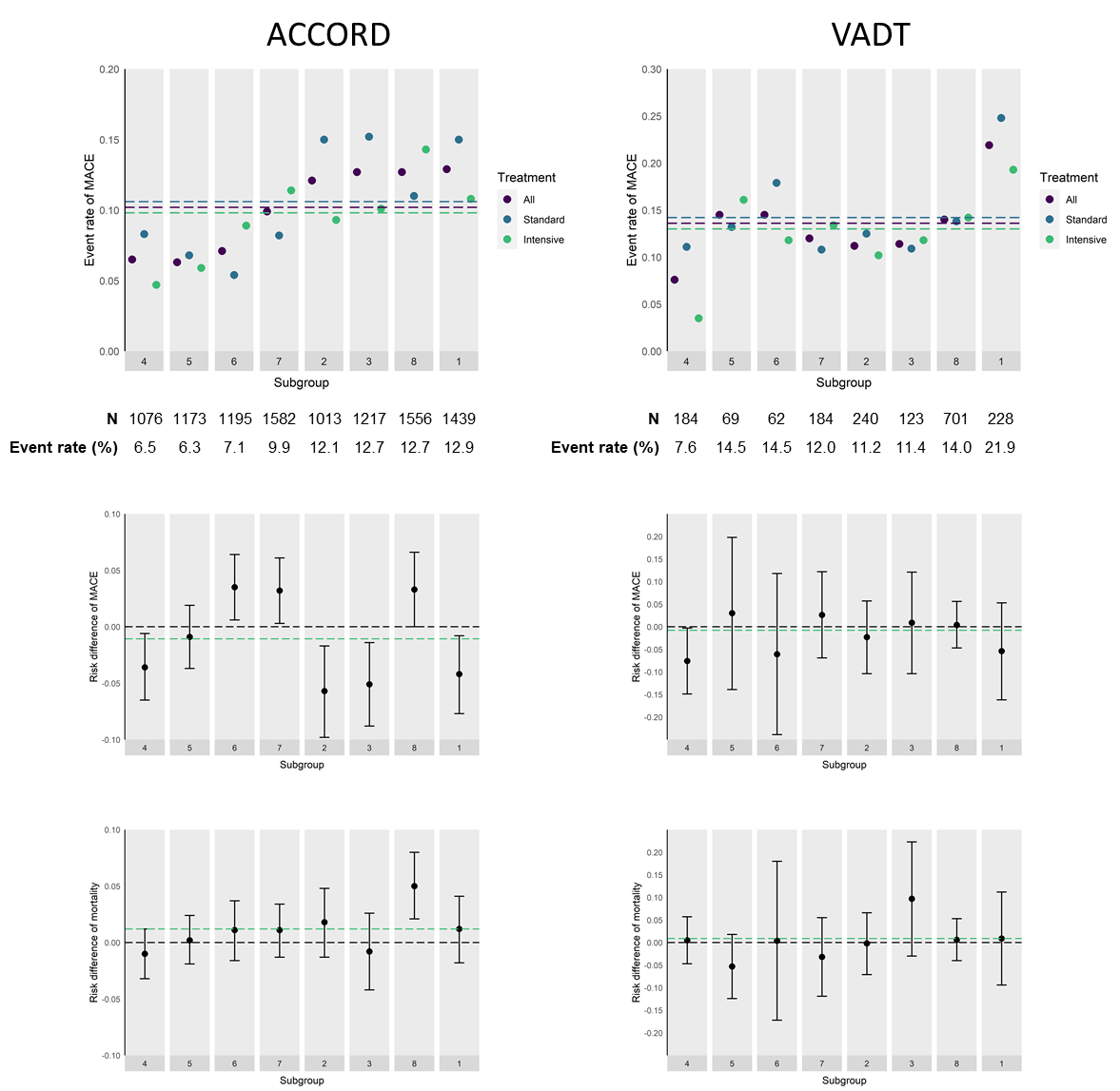
**

**Supplemental Figure 2.** Comparison of subgroup effects based on subgroup-specific rates of major adverse cardiovascular events (MACE) stratified by study (ACCORD on the left, VADT on the right). Subgroups were ordered from left to right by event rates in pooled data including both ACCORD and VADT studies. Top panels show event rates in each subgroup across both treatment arms (“All”, purple), among those randomized to standard glycemic control (“Standard”, blue), and among those randomized to intensive glycemic control (“Intensive”, green). Dotted lines show the event rates of MACE in the full sample (purple), in those randomized to standard glycemic control (blue), and in those randomized to intensive glycemic control (green). Middle panels show risk differences of MACE associated with standard versus intensive glycemic control with subgroups ordered from left to right by increasing MACE event rates in the full sample including pooled data from both studies. Positive risk differences reflect higher MACE and negative risk differences reflect lower MACE in intensive glycemic control compared to standard glycemic control. Bottom panels show risk differences of all-cause mortality associated with standard versus intensive glycemic control with subgroups ordered from left to right by increasing MACE event rates in the full sample including pooled data from both studies. Positive risk differences reflect higher mortality and negative risk differences reflect lower mortality in intensive glycemic control compared to standard glycemic control. Green dotted lines in the middle and bottom panels show the average treatment effect of intensive versus standard glycemic control in each study – risk difference of MACE (middle panels) and of all-cause mortality (bottom panels).

**Supplemental Table 3.** Risk differences of mortality between intensive and standard glycemic control arms in in eight subgroups.

|  | | Subgroup 1 | Subgroup 2 | Subgroup 3 | Subgroup 4 | Subgroup 5 |  | Subgroup 6 | Subgroup 7 | Subgroup 8 |
| --- | --- | --- | --- | --- | --- | --- | --- | --- | --- | --- |
| **Mortality** | **ACCORD + VADT**  *Risk difference*  *(95% CI)* | 1.2%  (-1.7, 4.1) | 1.4%  (-1.4, 4.2) | 0.3%  (-3.1, 3.6) | -0.8%  (-2.8, 1.2) | -0.1%  (-2.1, 2.0) |  | 1.2%  (-1.5, 3.8) | 0.5%  (-1.8, 2.8) | 3.6%  (1.1, 6.1) |
|  | **ACCORD**  *Risk difference*  *(95% CI)* | 1.2%  (-1.8, 4.1) | 1.8%  (-1.3, 4.8) | -0.8%  (-4.2, 2.6) | -1.0%  (-3.2, 1.2) | 0.2%  (-1.9, 2.4) |  | 1.1%  (-1.6, 3.7) | 1.1%  (-1.3, 3.4) | 5.0%  (2.1, 8.0) |
|  | **VADT**  *Risk difference*  *(95% CI)* | 0.9%  (-9.4, 11.2) | -0.2%  (-7.1, 6.6) | 9.7%  (-3.0, 22.3) | 0.5%  (-4.7, 5.7) | -5.3%  (-12.4, 1.8) |  | 0.4%  (-17.2, 18.0) | -3.2%  (-11.9, 5.5) | 0.6%  (-4.0, 5.3) |

**Contributors to the VADT Study**

**Study Co-chairs:**

C. Abraira, Miami Veterans Affairs (VA) Medical Center, Miami.

W.C. Duckworth, Carl T. Hayden VA Medical Center, Phoenix, AZ.

**Miami Study Co-chair’s Office:** C. Paul, D. Arca, L. Cason, R. Martinez Zolotor, L. Williams.

**Phoenix Study Co-chair’s Office:** S.L. Collier, N. Ahmed, A. Boyd.

**Hines VA Cooperative Studies Program (CSP) Coordinating Center:**

D. Reda, Director

T. Moritz, study biostatistician

R. Anderson, subprotocol biostatistician

M.E. Vitek, quality assurance specialist

T. Paine, national study coordinator

L. Thottapurathu, statistical programmer

P. Luo, subprotocol statistical programmer

K. Bukowski, database programmer

D. Motyka, database programmer
V. Barillas, statistical assistant

R. Brown, statistical assistant

B. Christine, statistical assistant

L. Anfinsen, statistical programmer

M. Biondic, database programmer

R. Havlicek, statistical assistant

J. Kubal, national study coordinator, statistical assistant

M. McAuliffe, statistical assistant

M. McCarren, study biostatistician

M. Rachelle, statistical assistant

L. Rose, national study coordinator

J. Sacks, subprotocol biostatistician

T. Sindowski, statistical assistant

J.Thomas, national study coordinator

C. Zahora, national study coordinator

**CSP Coordinating Center, Albuquerque, NM:**

M.R. Sather, Director

S. Warren, study pharmacist

J. Day, pharmaceutical project manager

J. Haroldson, study pharmacist.

**Executive Committee:**

C. Abraira, W. Duckworth, S.N. Davis, N. Emanuele, S. Goldman, R. Hayward, J. Marks, T. Moritz, P. Reaven, D. Reda, S. Warren, F. Zieve, W. Wendell, J. Haroldson, P. Harper, W.G. Henderson, R.R. Henry, M.S. Kirkman, M. McCarren, J. Sacks.

**Data and Safety Monitoring Committee:** J. Gavin, E. Chew, B. Howard, T. Karrison, I.V. Pacold, D. Seigel, F. Vinicor, B. Massie, consultant.

**End-Points Committee:** S. Goldman, S. Rapcsak, G. Sethi, M. Sharon, H. Thai, K. Zadina, J. Christensen, D. Morrison, P. Spooner, A. Westerband.

**Consultants:** B. Materson, E. Brinton, R. Klein, J.A. Colwell, E.J. Schaefer, C.S. Gass.

**Central Laboratories:**

*C-peptide:* D.A. Ehrmann, P. Rue

*Biochemistry*: E.J. Schaefer, J.R. McNamara

*MAVERIC Core Laboratory*: M. Brophy, D. Humphries, D. Govan, L. McDonnell, L. Carlton, Y. Weng

*Cost-Effectiveness:* R.A. Hayward, S. Krein

*Electrocardiography:* S. Goldman, K. Zadina

*Fundus Photograph Reading Center*: M. Davis, Director; K. Glander, project coordinator.

**The following investigators and sites participated in the study:**

*Charleston, SC:* J. Soule, S. Caulder, C. Pittman, O. Alston, R.K. Mayfield, G. Moffitt, J. Sagel, F. Sanacor, E. Ganaway

*Miami:* J. Marks, L. Okur, L. Jones, H. Florez, D. Pfeifer, L. Samos, A.L. Taylor

*Lyons/East Orange, NJ:* M.B. Zimering, A. Sama, F. Rosenberg, H. Garcia, N. Ertel, L. Pogach, J.J. Shin, F. Caldarella, C. Carseli, M. Shah

*Fresno, CA:* P. Ginier, G. Arakel, Y. Fu, D. Tayloe, J.E. Allen, E. Fox, P.G. Hensley

*Hines, IL:* N. Emanuele, K. Kahsen, P. Linnerud, L. Agrawal, N. Azad

*Houston:* M. Marcelli, G.R. Cunningham, N.M. Nichols, E. Cordero, R. Hijazi, F. Roman, P. Datta, M. Garcia Touza

*Indianapolis:* A. Lteif, K.L. Moore, C. Lazar-Robinson, S. Gupta, M.S. Kirkman, M. Mendez, Z. Haider, L. Risley

*Lexington, KY:* D. Karounos, L. Barber, J. Hibbard, J.W. Anderson, L.R. Reynolds, J. Carlsen, R.W. Collins, A. Ehtisham

*Long Beach, CA:* M.L. Kashyap, B. Matheus, T. Rahbarnia, A.N. Vo, N. Downey, L. Fox, R.M. Gonzales, C.D. Meyers, S. Tavintharan

*Minneapolis:* F.Q. Nuttall, L. Cupersmith, K. Dardick, L. Kollman, A. Georgopoulos, C. Niewoehner; *N ashville:* S.N. Davis, P. Harper, D. Davis, J. Devin, A. Marney, J. Passyn-Dunn, J. Perkins, J. Stafford, A. Powers, L. Balch, P. Harris

*Omaha, NE:* R.J. Anderson, D. Dunning, S. Ludwig, M. Vogel, C. DeSouza, R. Ecklund, S. Doran, C. Korolchuk, M. McElmeel, S. Wagstaff; *Phoenix, AZ:* P. Reaven, B. Solie, J. Matchette, C. Meyer, S. Vela, N. Aslam, E. Brinton, J. Clark, A. Domb, L. McDonald, L. Shurtz; *Pittsburgh:* R.H. Rao, J.N. Beattie, C. Franko, F.R. DeRubertis, D. Kelly, M. Maser, J. Paul

*Richmond, VA:* F. Zieve, S.J. Clark, A. Grimsdale, S. Fredrickson, J. Levy, D. Schroeder

*Salem, VA:* A. Iranmanesh, B. Dunn, D. Arsura, C. Kovesdy, S. Hanna, A. Iranmanesh, C. Florow, F. Remandaban, E. Smith; *San Diego, CA:* R.R. Henry, M. Keller, V. Aroda, C. Choe, S. Edelman, A. Gasper, D. MaFong, S. Mudaliar, D. Oh, R. Bandukwala, A. Chang, S. Chaudhary, S. Chinnapongse, L. Christiansen, N. Chu, D. Kim, M. Lupo, C. Manju, R. Plodkowski, R. Sathyaprakash, J. Wilson, J. Yu, G. Macaraeg, S. Tornes

*San Antonio, TX:* R. DeFronzo, L. Johnson, K. Cusi, D. Tripathy, M. Bajaj, J. Blodgett, S. Kayshup, M.H. Vasquez, B. Walz, T. Weaver

*San Juan, Puerto Rico:* J. Benabe, Z. Mercado, B. Padilla, J. Serrano-Rodriguez, C. Rosado, E. Mejias, T. Tejera, C. Geldrez, E. Gonzalez-Melendez, M. Natal, M. Rios Jimenez

*Tucson, AZ:* J.H. Shah, W.S. Wendel, L. Scott, L.A. Gurnsey, F.A. Kwiecinski, T. Boyden, M.G. Goldschmid, V. Easton.
